
JOURNAL INFORMATION
==============================
NLM Title Abbreviation: J Child Orthop
Journal ID: J Child Orthop
NLM Journal ID: 101313582

Journal of Children's Orthopaedics

ISSN: 1863-2521
EISSN: 1863-2548
Publisher: SAGE Publications

ARTICLE INFORMATION
==============================
PMCID: PMC4794451
PMID: 26984677
DOI: 10.1007/s11832-016-0716-x
Article ID: 716
Article version: 1
Subjects: Abstracts

EPOS 35th Congress Meeting

Electronic publication date: 2016 Mar 16
Print publication date: 2016 Apr
Volume: 10
Issue: Suppl 1
First page: 81
Last page: 89
Copyright: © The Author(s) 2016
License: This article is distributed under the terms of the Creative Commons Attribution 4.0 International License (http://creativecommons.org/licenses/by/4.0/), which permits unrestricted use, distribution, and reproduction in any medium, provided you give appropriate credit to the original author(s) and the source, provide a link to the Creative Commons license, and indicate if changes were made.
License URL: https://creativecommons.org/licenses/by/4.0/

issue-copyright-statement: © The Author(s) 2016

==============================

## PP1

### Splinting for the treatment of the pediatric trigger thumb: a pilot-study

**Sebastian Farr**[1], Werner Girsch[1]

[1]Orthopaedic Hospital Speising, Vienna, Austria

**Level-4**

**Other**
***Introduction*** Surgery remains the method of choice for the treatment of pediatric trigger thumbs for most pediatric orthopedic surgeons. However, some controversy still exists whether conservative treatment may be a useful option for this entity. We recently published a systematic review, which highlighted the superior efficacy of surgery versus splinting (95 vs. 67 % full IP-joint motion). The aim of this investigation was to review our own patient cohort with regards to success rates after splinting therapy. Given that the current literature on splinting is rarely stratified based upon disease severity, we furthermore sought to assess success rates depending on whether flexible or fixed trigger thumbs were present.
***Material and methods*** We performed a pilot-study including a total of 13 children (15 thumbs), who received a standardized treatment protocol including a custom made thermoplastic splint. Parents were advised to apply the splint during sleeping hours for a minimum of 3 months. Children were included regardless of their stage of the disease (flexible or fixed IP-joint flexion contractures; Watanabe types 1–3). Primary endpoint was conversion to surgery determined after chart review after a mean follow-up of 2.4 years (range 1.2–2.8 years). Paired t-tests were performed to compare the amount of pre- and post-splinting IP-joint flexion contractures.
***Results*** A total of 13 children with a mean age at initiation of therapy of 1.9 years (range 0.1–3.8 years) were included. The cohort consisted of nine left and six right thumbs, who showed Watanabe type 1 ($n = 5$), type 2 ($n = 6$), and type 3 ($n = 4$) features, respectively. Two patients (15 %) were lost to follow-up and their eventual outcome remains unknown. The splints were worn by the remaining cohort for a mean period of 7.3 months (range 1.2–14.4 months). During this period, the IP-joint contracture range decreased from a mean of 20.2° (range 0°–45°) to a mean of 11.1 degrees (range, 0°–45°); this decrease was, however, not statistically significant on analysis ($p = 0.157$). Splinting was successful for 4/5 (80 %) patients with Watanabe type 1, 3/5 (60 %) patients with Watanabe type 2, but only 1/3 (33 %) showing Watanabe type 3 thumbs, respectively. The overall success rate was 62 %.
***Conclusion*** Our results corroborate results in the current literature and indicate that splinting therapy is of only minor value for moderate and severe cases (Watanabe types 2 and 3). In these instances surgery should remain the method of choice to reliably correct the thumb flexion contraction. Nevertheless, splinting was beneficial for the majority of patients showing triggering symptoms only (Watanabe type 1). Larger clinical series focusing on splinting of especially mild cases are needed to fully clarify the value of this conservative treatment method.
***Significance*** Although surgery for trigger thumbs is generally fast and effective, our results indicate that splinting therapy might be a useful option for mild cases. If these results can be confirmed by other studies, surgery could be avoided for a relatively large amount of children which are affected by this condition.

## PP2

### Osteopenic features of hip joint in patients with cerebral palsy: a hospital-based study

**Myung Ki Chung**[1], Sang Young Moon[2], Soon-Sun Kwon[3], Chin Youb Chung[1], Kyoung Min Lee[1], Ki Hyuk Sung[1], Byung Chae Jo[1], In Hyeok Lee[4], Ki Jin Jung[5], Moon Seok Park[1]

[1]SNUBH, Kyungki, Republic of Korea; [2]Hallym University, Kangwon, Republic of Korea; [3]Ajou University, Kyungki, Republic of Korea; [4]Samsung Changwon, Changwon, Republic of Korea; [5]Soon Chun Hyang, Cheonan, Republic of Korea

**Level-3**

**Neuromuscular/Cerebral Palsy/AMC/MMC**
***Introduction*** We aimed to evaluate the bone mineral density of children and adolescents with cerebral palsy (CP) by assessing bone attenuation of the hip joint measured on computed tomography (CT).
***Material and methods*** Patients with CP aged <18 years who underwent 3-dimensional hip CT examination were analyzed. Mean bone attenuation of the acetabulum and femoral neck was measured on CT images and recorded as Hounsfield units. Differences in bone attenuation of the hip joint were analyzed and adjusted for affecting factors, such as age, sex, involvement (unilateral vs bilateral), hip instability, and gross motor function classification system (GMFCS) level.
***Results*** One hundred and twenty-six children and adolescents with CP and 86 normally developing subjects were included in this study. Average bone attenuation of the acetabulum and femoral neck in those with CP was significantly lower than those in the normally developing comparison group ($p < 0.001$), and decreased as GMFCS level increased ($p < 0.001$). It is noteworthy that average bone attenuation of the hip joint was significantly affected by migration percentage (acetabulum, $p = 0.047$; femoral neck, $p < 0.002$).
***Conclusion*** Average bone attenuation of the acetabulum and femoral neck was significantly affected by both GMFCS level and severity of hip instability.

## PP3

### Use of glenoid inclination angle for the assessment of unilateral congenital high scapula

**Naoyuki Nakamura**[1], Yutaka Inaba[2], Jiro Machida[1], Tomoyuki Saito[2]

[1]Kanagawa Children's Medical Center, Yokohama City, Japan; [2]Yokohama City University, Yokohama City, Japan

**Level-4**

**Tumours**
***Introduction*** Sprengel's deformity is a congenital anomaly in which one or both scapulae are positioned higher than normal. For shoulder joint dysfunction associated with Sprengel's deformity, conservative therapy is generally ineffective. In surgical procedures, favorable outcomes are achieved in terms of improvement in the range of abduction of the upper limb. The outcomes vary mainly because of cosmetic aspects including the size of the surgical scars, winging of the scapula, and relief of the trapezius muscle. However, little has been reported on the motion of the scapula in Sprengel's deformity.
***Material and methods*** Fourteen patients with unilateral congenital high scapula underwent the Woodward procedure from 1986 to 2004. The median age of the patients at the time of surgery was 4.4 years and the median follow-up duration was 8.8 years. The range of motion (ROM) of the upper limb, scapular lowering, and scapular abduction [via the glenoid inclination angle (GIA)] were measured preoperatively and at the final visit.
***Results*** The ROM of the shoulder joint (flexion and abduction) improved from 100° preoperatively to 160° at the final visit. The positional differences were reduced from 25.0 to 8.5 mm and from 13.5 to 6.8 mm in the inferior scapular angle and lower glenoid margin, respectively. The GIA improved from −15.5° to 0° with the upper limb in a neutral position and from 5° to 31° with the upper limb in maximum abduction.
***Conclusion*** The range of motion of the shoulder and GIA improved at the final visit. There was a positive correlation between the maximum abduction angle of the upper limb and GIA at the final visit.
***Significance*** The GIA, appears to be a simple and precise indicator of range of movement: it allowed us to confirm that improvement in scapular abduction was correlated with the range of upper limb abduction at the final visit.

## PP4

### Associated injuries in skeletally immature children with pelvic fractures

**M. Kareem Shaath**[1], Kenneth Koury[1], Peter Gibson[1], Mark Adams[1], Michael Sirkin[1], Mark Reilly[1]

[1]Rutgers, NJMS, Newark, USA

**Level-4**

**Upper and lower extremity trauma**
***Introduction*** Pediatric pelvic fractures are rare injuries with a reported incidence of 1 per 100,000 children per year. Such injuries result from high-energy mechanisms that warrant an extensive workup for associated injuries. We performed a retrospective study to review concomitant injuries in children who suffered a pelvic fracture and have an open triradiate cartilage.
***Material and methods*** Using a trauma database, all pediatric pelvic fractures presenting to the authors' institution were extracted. Radiographs and CT scans were reviewed to ensure that tri-radiate cartilages were not fused and the pelvic injuries were classified using the Modified Torode Classification. Epidemiologic data included age, race, gender time of presentation, mechanism of injury, Glasgow Coma Scale (GCS), Injury Severity Score (ISS), Abbreviated Injury Score (AIS), units of blood transfused, length of hospitalization, need for surgical orthopedic intervention, and disposition. Associated injuries were classified as injuries to the head/neck region, injuries to face, injuries to chest, injuries to abdomen, and injuries to extremities.
***Results*** Sixty patients met the inclusion criteria and their average age was 8.3 years. Eleven patients required transfusions. Hospital stays ranged from 1 to 27 days with an average of 10.6 days and there were no fatalities. The most common mechanism of injury was a vehicle striking a pedestrian. There were no significant correlations between GCS, ISS, and AIS. All 60 children (100 %) suffered additional extremity injuries. Nineteen patients required surgical orthopedic intervention and six required operative stabilization of the pelvis. Patients who were struck by a motor vehicle were more likely to have multiple pelvic fractures ($p < 0.05$). Patients with multiple pelvic fractures were also more likely to require orthopaedic surgical intervention and require a blood transfusion ($p < 0.05$). Patients who had type III-B or IV fractures were more likely to require a transfusion than patients with a type III-A fracture ($p < 0.05$). The 13 patients with concomitant acetabular fractures were no more likely to have surgical orthopaedic intervention ($p = 0.83$).
***Conclusion*** Patients sustaining pelvic fractures to an immature pelvis are likely to have additional injuries. The high energy required to fracture the immature pelvis can lead to several severe or critical injuries which may be fatal or disabling if not diagnosed in a timely manner.
***Significance*** This is the only study to specifically focus on injuries associated with pelvic fractures in patients with open triradiate cartilage.

## PP5

### New parameters for atlantoaxial instability in children with down syndrome

**Naoyuki Nakamura**[1], Yutaka Inaba[2], Jiro Machida[1], Tomoyuki Saito[2]

[1]Kanagawa Children's Medical Center, Yokohama City, Japan; [2]Yokohama City University, Yokohama City, Japan

**Level-3**

**Spine**

***Introduction*** Last year, we developed the C1/4 SAC ratio and the C1 inclination angle, which are noninvasive and reliable radiographic measurements of atlantoaxial instability (AAI) in children with Down syndrome (DS). The present study aimed to investigate the meaning of normal and abnormal values of these parameters by expanding the study population.

***Material and methods*** We recruited 315 children with DS, including 14 surgical cases and 237 children whose cervical segments had no cervical abnormality, including adenoidism, neck pain, and croup, from August 2013 to March 2015. The C1/4 SAC ratio, C1 inclination angle, atlas-dens interval (ADI), and space available for spinal cord (SAC) were measured in children with DS, and the C1/4 SAC ratio and C1 inclination angle were measured in non-DS cases. We obtained magnetic resonance (MR) images of 54 cases and measured the C1/4 cord ratio and the C1 cord/C4 dural sac (dsac) ratio, which were defined as the ratios of the anteroposterior diameter of the spinal cord at the C1 level to the anteroposterior diameter of the spinal cord and of the dural sac at the C4 level on T2 weighted MR images, respectively.

***Results*** The average C1/4 SAC ratios in surgical cases with DS, non-surgical cases with DS, and non-DS cases were 0.63, 1.16, and 1.27, respectively, and the C1 inclination angles were −3.1°, 15.7°, and 17.7°, respectively. The average ADI and SAC in surgical cases with DS and non-surgical cases with DS were 9.8 and 4.3 mm, and 11.1 and 18.9 mm, respectively. In cases with MR images, the average C1 cord/C4 dsac ratio and C1/4 cord ratio in surgical cases with DS and non-DS cases were 0.37 and 0.67, and 0.64 and 1.19, respectively.

***Conclusion*** The normal value of the C1/4 SAC ratio was estimated at approximately 1.2, and the C1 inclination angle was estimated at approximately 15°. The results of 0.67 for the C1 cord/C4 dsac ratio in non-DS cases and 0.63 for the C1/4 SAC ratio in surgical cases with DS may indicate cord compression by the C1 posterior arch in the neutral position in critical cases. It will be useful to know whether the C1/4 cord ratio is 1.19 in normal cases when evaluating cord atrophy of AAI in children with DS.

***Significance*** These new parameters may be of value when evaluating AAI in children with DS.

## PP6

### Is neurovascular compromise in complex limb reconstruction a risk with epidural analgesia in children?

**Julia Judd**[1], Michael Uglow[1]

[1]University Hospital Southampton, Southampton, UK

**Level-3**

**Other**

***Introduction*** The purpose of this study was to evaluate the incidence of neurovascular compromise including compartment syndrome in children whose post-operative pain relief was managed with an epidural, following application of an external fixator for the purposes of lower limb deformity correction and lengthening.

***Material and methods*** Between January 2001 and January 2014, a case series of patients undergoing external fixator management of their lower limb condition was reviewed. A retrospective medical record and chart review examined clinical data and included treatment condition, surgical procedure, prescription of epidural infusion, validated pain scores on day one and two post-operatively, neurovascular, urinary and specific epidural problems. Particular emphasis was made to identify neurovascular compromise.

***Results*** Eighty six patients with a total of 99 case episodes were included. A further four cases were excluded due to incomplete data, leaving 95 individual case episodes in 65 children. Eighty nine were treated with either a Taylor Spatial (TSF) or Ilizarov frame (IF) and 6 with a monolateral rail. The orthopaedic conditions included: relapsed club foot (42), leg length discrepancy (33), deformity correction (17), fractures (2) and one pelvic support osteotomy. The average age of the child was 11 years (range 4–18). The ratio of males to females was 34:31. Post-operative analgesia involved an epidural with a low concentration of local anaesthetic combined with an opiate agent. An intra-operative prescription of 0.25 %/0.5 % Bupivicaine (+/−Fentanyl, 2 mcg/kg) and a post-operative prescription of 0.15 %/0.125 % Bupivicaine (+/− Fentanyl) was prescribed. The epidural was usually in situ for 48 h post operatively (range 8–96 h) and was supplemented with Paracetamol, Ibuprofen and Codeine. Ibuprofen was only administered in the first 48 h due to its link to poor bone healing and since completion of this study the use of Codeine is contraindicated in children under the age of 12 years. No compartment syndromes occurred in the study period but there was one case of foot ischaemia resulting in dry gangrene and subsequent amputation of the little toe. The pain scores escalated rapidly during the second post-operative day despite a fully functional epidural.

***Conclusion*** Epidural analgesia is a very effective method of controlling post-operative pain and does not appear to mask the pain of ischaemia.

## PP7

### Relative lengthening of the femoral neck with distal transfer of greater trochanter in children and adolescents

**Stephan Tserovsky**[1], Raytcho Kehayov[1], Boyan Christov[1], Venelin Alexiev[1]

[1]University Hospital of Orthopaedics and Traumatology, Sofia, Bulgaria

**Level-3**

**Hip/DDH/Legg-Calvé-Perthes/SCFE**

***Introduction*** The aim of this study is to present the relative lengthening of the femoral neck with distal transfer of the greater trochanter during surgical dislocation of hip in cases of coxa vara, Kalamchi type III osteonecrosis after treatment of developmental dysplasia of the hip and coxa breva after Perthes' disease.

***Material and methods*** Between 2010 and 2014, nine transfers of high-standing greater trochanter with relative lengthening of the femoral neck were performed in children and adolescents. The mean patient age was 12.3 ± 2.47 years. The aetiology of the cases was: two following valgus osteotomies due to developmental coxa vara; two cases after Stulberg type IV avascular osteonecrosis and five cases with Kalamchi type III osteonecrosis after developmental dysplasia of the hip. To determine the height of the greater trochanter, and follow up the results we used our radiographic classification, evaluating the articulo-trochanteric index. All patients were treated with surgical dislocation of the hip according to Ganz surgical technique. The greater trochanter was retracted anteriorly. Thereafter a triangular shaped fragment, including the apophysis of the trochanter, just above the piriform fossa was cut. This was done from inside out and from distal to proximal, along the upper surface of the femoral neck to the lateral edge of the femur. The new formed part of the neck is partially modified to the base of the trochanter. The correct position of the greater trochanter was established under image intensification by placing K-wire tangential and perpendicular from the tip of the trochanter to the center of the femoral head. The fixation was done with two, rarely three cannulated 3.5 or 4.5 mm fully-threaded cortical screws.
***Results*** The mean follow-up was 16.44 ± 12.71 months (between 8 and 48 months). All patients achieved a statistically significant lengthening of the femoral neck with distal transfer of greater trochanter according to our classification ($p < 0.01$). We have no cases of avascular necrosis, nonunion of the trochanter and ectopic ossification. According to classification of Heyman and Herndon, four excellent and five good outcomes were established. In one case we made overcorrection, without impeding the functional outcome.
***Conclusion*** Surgical dislocation of the hip with cutting triangular shaped fragment allows secure protection of feeding vessels and full bone modeling under direct control. Recurrences are practically impossible due to the apophysis removal. The fully-threaded cortical screws provide stable osteosynthesis and the possibility of early weigh-bearing with aiming devices.

## PP8

### The modified lateral pillar classification: interobserver agreement and value as a predictor of final radiological outcome in Perthes disease

**Stefan Huhnstock**[1], Terje Terjesen[1], Else Merckoll[1], Anthony Catterall[1], Ola Wiig[1]

[1]Oslo University Hospital, Rikshospitalet, Oslo, Norway; [2]Royal National Orthopaedic Hos, London, UK

**Level-3**

**Hip/DDH/Legg-Calvé-Perthes/SCFE**
***Introduction*** Herring et al. originally described a reliable classification to predict the prognostic severity of the disease at the fragmentation stage. They refined the original classification in 2004, introducing a borderline B/C group in order to increase the accuracy of the classification. The purpose of our study was to assess the interobserver agreement and prognostic value of the modified lateral pillar classification.
***Material and methods*** We identified 139 patients who had been treated for Perthes disease at our institution from 1950 until 1984 and who had anteroposterior and frog-leg radiographs of the hips at diagnosis, 1- and 5-year follow-up. We randomised 50 patients and excluded patients who had reached early reossification at diagnosis ($n = 3$) and those with bilateral involvement ($n = 5$). There were 38 boys and 4 girls with an average age of 6.5 years at diagnosis (SD 1.9). 37 children were treated non-operatively and 5 with varus osteotomy.
Five observers classified the radiographs on two separate sessions according to the Catterall classification, Herrings original and modified lateral pillar classification. Stulberg's modified 3-group classification was applied at 5-year follow-up. Interobserver agreement was analysed using weighted kappa statistics and values were interpreted as follows: below 0.20 poor agreement, 0.21–0.40 as fair, 0.41–0.60 as moderate, 0.61–0.80 as good and over 0.80 as very good agreement. We defined for each patient the classifications which had been assigned by the majority of the observers. The prognostic value of the "true" Catterall, the modified and the original lateral pillar classification was assessed in all non-operatively treated patients in a cross table analysis with "true" Stulbergs 3-group classification as outcome variable. Gamma statistics for ordinal variables were used and the values interpreted as follows: below 0.24 no association, 0.25–0.49 weak; 0.50–0.74 moderate and values over 0.75 strong association.
***Results*** All classifications had moderate interobserver agreement (kappa 0.41–0.48). Herring's original lateral pillar classification was strongly associated with Stulberg's 3-group classification (Gamma 0.87, $p < 0.001$). The modified lateral pillar and Catterall classification showed moderate association (Gamma 0.69, $p = 0.002$ and 0.53, $p = 0.008$).
***Conclusion*** The supplement of the borderline B/C group to the original lateral pillar classification system did not increase the interobserver agreement or the prognostic value in our study.
***Significance*** We found that the original 3-group Herring classification was easier to use, it was as reliable and even had a higher prognostic value than its 4-group modification.

## PP9

### Selective ultrasound screening for DDH: what could we miss?

**Ambra Galla**[1], Elena Marich[2], Carlotta Pari[2], Giada Salvatori[2], Anna Brandolini[2], Daniele Barbaro[2], Leo Massari[2]

[1]"Marino Ortolani" Centre, Orthopedic Unit/ University Hospital S.Anna, Ferrara, Italy; [2] University Hospital S.Anna, Ferrara, Italy

**Level-2**

**Hip/DDH/Legg-Calvé-Perthes/SCFE**
***Introduction*** Over the past 20 years, there has been lively debate as to whether or not to screen for DDH and if you do, which is the better strategy: clinical with or without universal or selective ultrasound scans. The aim of our study was to examine the cases referred to our Institute for the diagnosis and management of DDH in order to discover what Graf types were identified on ultrasound scanning in the children without risk factors or clinical signs of DDH.
***Material and methods*** From 01/01/2000 to 31/12/2014, personal data, delivery presentation, family history of DDH, clinical findings at the time of referral, and clinical and ultrasonographic findings at the first visit were registered for each infant who presented to our centre. Of all the babies, only those without family history, without clinical findings positive for DDH and without breech presentation were extracted; of this restricted group we studied clinical and sonographic findings at our centre, and estimated the absolute value and the rate of every sonographic type.
***Results*** From 01/01/2000 to 31/12/2014, 19,427 babies were referred to the "Marino Ortolani" centre for diagnosis and treatment of DDH. Of these, 536 were excluded because previously treated or because of incomplete findings. Of 18,891 remaining babies, 12,888 did not have a family history, nor clinical findings positive for DDH nor a breech presentation. In this group of infants we found, at the first visit to our centre, 65 babies with clinical findings positive for DDH. Of 12.823 babies completely negative for family history and/ or breech presentation and/or clinical findings for DDH, 5060 were type 1A, 6926 were type 1B, 334 type 2A+, 213 type 2A−, 161 type 2B, 70 were type2C, 56 were type 2D, 3 were type 3 and none were type 4.

***Conclusion*** In our experience a selective ultrasonographic screening for DDH should imply a considerable number of misdiagnosed dysplasias and a few dislocations. In an expert hand the clinical exam should detect all the type 4 hips.
***Significance*** More studies are needed to clarify the role of clinical exam and ultrasonography in relation to screening for DDH. Both methods needs thorough training to achieve good mastery; if we ignore it, we could miss something whatever strategy we use.

## PP10

### Are sensitivity and specificity of clinical examination for DDH really low?

**Ambra Galla**[1], Elena Marich[2], Carlotta Pari[2], Giada Salvatori[2], Anna Brandolini[2], Daniele Barbaro[2], Leo Massari[2]

[1]"Marino Ortolani" Centre, Orthopaedic Unit/ University Hospital S.Anna, Ferrara, Italy; [2]University Hospital S.Anna, Ferrara, Italy

**Level-2**

**Hip/DDH/Legg-Calvé-Perthes/SCFE**
***Introduction*** A positive clinical examination is considered by most researchers as the main diagnostic sign of DDH in newborns. Nevertheless, sensitivity and specificity of the examination is reported as low in several studies. The aim of our study was to verify, in our cases, the sensitivity and specificity of clinical examination performed by paediatricians in maternity units and by expert orthopaedic surgeons.
***Material and methods*** We examined the clinical and ultrasonagraphic findings of every, consecutive infant at their first visit following referral to our centre from 1/1/2000 to 31/12/2014. Personal data, presentation at delivery, family history of DDH and clinical findings at the time of referral were registered for every infant. Afterwards we calculated sensitivity and specificity of clinical examination performed outside by paediatricians and within our centre, relative first to all the sonographic types of hip problems (2A-4) and with subsequent analysis looking only at types 3 and 4.
***Results*** During the time period of the study, 19,427 babies were referred to the Marino Ortolani centre for diagnosis and management of DDH. Of these, 536 were excluded because of previous treatment or incomplete data. Of 18.891 remaining cases, after sensitivity and specificity computation, a noticeable difference was detected between clinical examination performed by paediatricians in a maternity unit and by a paediatric orthopaedic surgeon at the Marino Ortolani Centre. Senstivity and specificity were higher when considering only type 3 and 4 hips as pathological.
***Conclusion*** When performed by a well trained and expert physician, clinical examination has a high sensitivity and specificity to detect DDH. Both sensitivity and specificity of clinical examination are higher when only type 3 and type 4 hips according to Graf are considered.
***Significance*** A positive clinical examination is the main diagnostic sign of DDH in newborns indicating a pathological Graf type 3 or 4 hip.

## PP11

### Plate fixation after correction of limb length discrepancy (LLD) with distraction osteogenesis method

**Nikita Khusainov**[1], Dmitry Buklaev[2], Sergey Bat'kin[2]

[1]Scientific Research Institute for Pediatric Orthopedics, n. a. H. I. Turner, Saint-Petersburg, Russian Federation; [2]Scientific Research Institute, Saint-Petersburg, Russian Federation

**Level-4**

**Foot and ankle**
***Introduction*** Our aim was to evaluate results of plate fixation for osteosynthesis of the zone of distraction regenerate after completion of the distraction period.
***Material and methods*** 32 patients with LLD were treated using Ilizarov frame and "MEFiSTO" device. After the completion of the distraction period, plate fixation with locking compression plates was performed along with frame removal. Patients were encouraged to stand, in orthoses, early after in the post-operative period.
***Results*** In two cases, stability of the fixation was lost with varus deformity of the femoral regenerate. In one other case, there was slow formation of the regenerate followed by valgus—recurvatum deformity of the tibial. Stable fixation of the zone of distraction regenerate was achieved in all the remaining cases.
***Conclusion*** Fixation after correction of LLD with distraction osteogenesis method by using LCP plates provides adequate stability and allows early removal of the ex-frame with small rate of complications.
***Significance*** Plate fixation after distraction completion in LLD correction allows the surgeon to decrease duration of frame fixation and total duration of treatment. Also a decrease in complications such as osteoporosis, infection, regenerate fractures was noted. It can be used when intamedullary nailing can not be applied. A negative aspect of this method is the necessity for hardware removal after consolidation.

## PP12

### Surgical treatment of equinoplanovalgus deformity in children with cerebral palsy

**Paviel Rakhman**[1], Oleksandr Korolkov[1]

[1]SE "Sytenko Institute of Spine and Joint Pathology", Kharkiv, Ukraine

**Level-3**

**Neuromuscular/Cerebral Palsy/AMC/MMC**
***Introduction*** Foot deformity in children with cerebral palsy are common. The natural history of these foot deformities is very variable and very unpredictable in children in different age groups. We aimed, in the current study, to compare the effectiveness of different types of surgery in children with cerebral palsy.

***Material and methods*** The treatment of 96 patients, aged 2–18 years, was studied: bilateral pathology was observed in 72 % of cases. Assessment of severity of the foot deformities before and after treatment was carried out according to statopodography, plantography, X-rays and electromyography. The patients were divided into age groups. In form: hemiparetic—24.2 %, double hemiparesis—14.5 %, spastic diplegia—37.1 %, hyperkinetic form—9.68 %, atonic-astatic—6.5 %, mixed form—8.1 %. According to the classification GMFCS, in the study group 12 %—I level, 22 %—level II, 37 %—III 26%—IV level, 13%—V level. The mean follow-up was 5.6 years.
***Results*** In the first group: 14 cases achieved correction with conservative treatment. Percutaneous Achilles tenotomy or tendon lengthening was performed in 34 patients, including 11 patients who underwent a tenodesis of the posterior tibial muscle tendon with the transposition of the anterior tibial muscle under the scaphoid bone. In 12 cases after lengthening of the Achilles tendon we took hold back capsulotomy of ankle and subtalar joints. We achieved early activation on the 3–5 day after surgery, but with fixation in cast for 3–4 weeks.
In the second group, in 31 cases we applied the method of subtalar arthroereisis and in 90 % of cases a percutaneous Achilles tenotomy was performed. In 75 % of cases, a shortening tenodesis of the posterior tibial muscle took place and in 22 % the surgeons also performed a proximal wedge osteotomy of the first metatarsal bone with fixation by compression screws. In the third group, in nine cases we performed hindfoot corrective arthrodesis and in five cases and Evans calcaneal osteotomy, and a further five had medialization and displacement of calcaneus. Talo-calcaneal angle in all groups before treatment $51^{\circ} \pm 6^{o}$, after $37^{\circ} \pm 5^{\circ}$, hindfoot valgus—before $16^{\circ} \pm 7^{\circ}$, after treatment $0^{\circ} \pm 4^{\circ}$.
***Conclusion*** Assessment of equinoplanovalgus foot deformity in children with CP, allows you to develop a differentiated approach to the treatment based on the patient's age and the degree of deformation.
***Significance*** Development algorithm of treatment of the equinoplanovalgus foot deformity in children with CP.

## PP13

## Comparative evaluation of a positive-locking antirotation mechanism in the design of telescopic nails for growing patients with OI or other rare skeletal conditions

**Frank Schiedel**[1], Jens Lakomy[1], Bjoern Vogt[1]

[1]Muenster University Hospital, Muenster, Germany

**Level-4**

**Basic science**
***Introduction*** Telescopic nails are commonly used for treating fractures in patients with OI or CPT. Poor rotational stability with current telescopic nails leads to inadequate postoperative bone healing and hypertrophic pseudarthroses. Rotational stability is often only achieved through external stabilization (casts or orthoses). However, this immobility leads to osteopenia, with negative effects on the course of healing; again increasing the risk of subsequent fracture. The aim of the development and biomechanical study described was to achieve rotational stability without external stabilization, using a new nail design and a modified implantation technique to establish safe and also rotationally stable proximal and distal fixation of the two telescopic nail components within the bone, while also avoiding soft tissue damage.
***Material and methods*** Three prototypes for a positive-locking adapted telescopic intramedullary nail (PLATIN) were developed consisting of two components, a tube and a rod. The female part was round on the outside, with a uniform hexagonal cross-section on the inside throughout its entire length. The male part had a uniform hexagonal cross-section along its entire length. The female and male parts were manufactured to a tolerance of approximately 0.003 mm, allowing frictionless sliding of the male part within the female part. This mechanism allows rotationally stable telescoping of the two components, with almost no free play. At one end of the female part, a thread with a large thread depth and 2.5 turns was welded on. Halfway down the length of the thread, there was a bilaterally conical cross-hole. A 20-mm long cylinder matching the outer diameter of the female part was welded to one end of the male part. The cylinder ended in a tip traversed by three cutting blades. Two cross-holes were drilled into the cylinder at a distance of 13 mm apart, identical in type to those in the female part. The two cross-holes allow variable placement of the locking wire, depending on the position of the end of the nail relative to the epiphyseal plate. The cross-holes in the female and male parts were in alignment. In a series of biomechanical tests, the prototypes were compared with two Fassier–Duval telescopic nails, which represent the clinical standard. Axial pressure, torsion and four-point bending measurements were carried out in a materials testing machine, with the telescopic nails implanted into composite bone. Specifically, the force required for telescoping, as well as torsional stiffness and bending stiffness, were investigated. Taking into account differences that were inherent to the materials, the prototypes showed similar results in the pressure tests and four-point bending tests.
***Results*** In the axial compression test, it was found that the prototypes required 2.8–7.1 times more force for telescoping in comparison with the FD telescopic nails. The Mann–Whitney $U$ test showed significant differences ($P < 0.0001$; $U = -9.860$ and $-7.488$) with regard to axial force between PR30 and FD32 and between PR50 and FD48. In comparison with the corresponding FD nails, the torsion test showed 20–15 times more torsional stiffness in the prototypes. The Mann–Whitney $U$ test showed significant differences in torque in the torsion test without failure between PR30 and FD32 and between PR50 and FD48 ($P < 0.0001$; $U = -20.120$ and $-25.764$).
***Conclusion*** This study was concerned with the development of an innovative intramedullary telescopic nail, with a special focus on establishing rotational stability and optimizing axial stability by improving bone anchorage. The test results show that the new design is potentially beneficial for therapy.
***Significance*** Potentially of clinical value in treating this difficult group of fractures.

## PP14

## Prediction of rebound phenomenon after removal of hemiepiphyseal staples in patients with idiopathic genu valgum deformity

Seungcheol Kang[1], Jae Young Kim[1], **Il Yeong Hwang**[1], Soo-Sung Park[1]

[1]Asan Medical Center, Seoul, Republic of Korea

**Level-3**

**Other**
***Introduction*** Although the rebound phenomenon (RbP) after temporary hemiepiphysiodesis is frequently reported, there have been few studies investigating its prediction. The purpose of our present study was to investigate practical guidelines predicting the RbP (frequency and severity) after implant removal. We (1) evaluated the independent predictors of the RbP, (2) generated a predictive grouping model using those independent predictors, and finally (3) compared and confirmed the differences in the incidence of RbP among the groups.
***Material and methods*** We targeted 37 subjects with idiopathic genu valgum deformity, treated with hemiepiphyseal stapling, and with a remaining growth period of more than 6 months at staple removal. All patients were followed up until skeletal maturity or for more than 2 years after staple removal. A predictive model for the RbP was generated by using recursive partitioning analysis (RPA) with the independent predictive factors elicited from multivariate analyses.
***Results*** On multivariate analyses, the rate of correction, body mass index (BMI), multiplier value, and initial valgus angle were significantly associated with the RbP. On RPA using those characteristics, the rate of correction and BMI successfully divided the patients into three predictive groups with significant differences in the frequency and severity. Patients with a rate of correction $\geq$8.5°/year showed the most frequent and severe RbP (incidence: 79 %, mean: 4°), whereas patients with a rate <8.5°/year showed less (if BMI <21 kg/m$^2$, 43 %, 2°) or no (if BMI $\geq$21 kg/m$^2$, 0%, 0°) RbP.
***Conclusion*** The activity of the physis (rate of correction) and its suppression by relative weight (BMI) seem to be primarily associated with the occurrence of the RbP. Using RPA, the rate of correction and the BMI successfully divided our study patients into three predictive groups that showed significant differences in the frequency and severity of the RbP.
***Significance*** This is the first study to evaluate the predictive factors for the RbP after temporary hemiepiphysiodesis among patients with idiopathic genu valgum. Although further studies are needed, practical guidelines for the compensation for RbP are suggested by our present findings.

## PP15

### Ultrasound study of the hips of preterm babies separates genetic factors from mechanical factors that shape the neonate hip. Etiologic implications in the study of developmental dysplasia of the hip

**Panagiotis Samelis**[1], Nikolaos Anoua[1], Christos Nikakis[1], George Mitrogiannis[1], Charalampos Lalos[1], Apostolos Konstantinou[1], Christos Zambakidis[1], Ioannis Kapetanakis[1]

[1]P&A Kyriakou Children's Hospital of Athens, Athens, Greece

**Level-4**

**Hip/DDH/Legg-Calvé-Perthes/SCFE**
***Introduction*** The development of the acetabulum during gestation follows a defined timetable. The hip joint appears at 4th week of the embryonic life. At 11th week the femoral head is almost completely surrounded by the acetabulum. Ongoing development of the fetal hip results in progressive uncovering of the femoral head in favour of increased hip mobility. The last days of gestation affect the shape of the acetabulum significantly due to high pressures exerted on the foetus in preparation for birth. The shape of the hips of preterm babies depends rather on genetic factors than on mechanical pressure of normal birth. A study of the intrauterine development of the hips of preterm babies might define the mechanical effect on the genetically determined development of the hip.
***Material and methods*** The hips of 43 preterm babies, gestation age 24–36 weeks, birth weight 540–2340 g, were studied clinically and ultrasonographically. Clinical testing of stability included the Barlow and Ortolani maneouvres and range of motion testing of the hips as well. The bony acetabular roof was measured ultrasonographically according to the Graf method (alpha angle).
***Results*** The alpha angle gestational age curve is described. A significant decrease of the alpha angle of the acetabulum with increased gestational age is observed. The alpha angle drops from 86° at the 24th week to 65° at the 36th week of fetal life. Otherwise healthy preterm babies have highly stable hips.
***Conclusion*** During the few last days of gestation, rising intrauterine pressure plays an important role on shaping the developing hips of the foetus. Most factors currently accepted to predispose to Developmental Dysplasia of the Hip, seem to result in plain direct pressure on the acetabular cartilage anlage, driving the evolving acetabular roof out of its normal balance, thus leading to a shallow acetabulum with or without a true dislocation of the hip. The postnatal hip continues to shape itself depending on external forces guided by the femoral head. Neonatal hip dysplasia may be the result of acute traumatic or chronic abnormal pressure on the foetal hip. Ultrasound monitoring of the alpha angle of the hips of preterm babies unlinks the genetically determined development of the acetabulum from mechanical pressure that normal birth inevitably exerts on the foetal hips. This knowledge may shed light on the aetiology of newborn hip dysplasia.
***Significance*** A method to unlink genetic and mechanical factors that shape the hip during gestation.

## PP16

### Navicular tenosuspension with anterior tibialis tendon (Young procedure) associated to calcaneo-stop for the treatment of paediatric flexible flatfoot: clinical and ultrasound study

**Elena Manuela Samaila**[1], Ingrid Bonetti[1], Bruno Magnan[1]

[1]University of Verona, Italy, Verona, Italy

**Level-4**

**Foot and ankle**
***Introduction*** Flexible flatfoot is one of the most common deformities in pediatric orthopedic clinics. It is characterized by an absence of the medial arch and a valgus position of the calcaneus. Arthroereisis procedures are designed to correct this deformity. Amongst these procedures, the calcaneo-stop is one with both biomechanical and proprioceptive properties. In the history of flatfoot surgery, there are many surgical procedures (eg. percutaneous achilles tendon lengthening and medial surgical procedures) that were first proposed as the only treatment. Many of these were abandoned because of an inadequate correction. Capsule-tendon plications of the medial arch have been used as combined procedures for TP insufficiency/laxity. Restoration of the talo-calcaneal alignment is the main goal and today the golden standard procedure for this is the subtalar arthroereisis (for us: calcaneo-stop). Our therapeutic algorithm for flatfoot in childhood is an arthroereisis associated with a navicular resection and plication of the posterior tibial tendon (Kinder-Gould) in the presence of a talo-navicular subluxation (Meary line broken at the talo-navicular joint) or a navicular tenosuspension with the anterior tibial tendon (AT) (Young) when there is a navicular-cuneiform subluxation (Meary line broken at the navicular-cuneiform joint).
***Material and methods*** From March 2001 to August 2014, 36 patients (54 feet) were treated with calcaneo-stop arthroereisis, percutaneous achilles tendon lengthening and Young's tenosuspension. 33 feet were symptomatic and 21 feet were asymptomatic. The Young procedure begins with a medial approach, isolation of the tibialis posterior tendon, navicular resection, isolation of the anterior tibial tendon; a tunnel is made in the navicular from dorsal to plantar and proximal to distal that is transformed into a groove. The anterior tibial tendon is driven into the groove and held with transosseous sutures and a tenodesis between anterior and posterior tibial tendon is performed. The procedure was performed bilaterally, as a staged procedure, in eighteen cases. Clinical assessment (Hindfoot AOFAS) and ultrasonography studies of the tibialis anterior tendon were performed in all patients.

***Results*** The average follow-up was 7.4 years (range 8 months–14 years). At follow-up, the outcome was satisfactory in 51 feet (94.5 %). No major or minor complications, were observed. In four cases the calcaneo-stop was removed for pain and low tolerance by the patient. In 28 cases (51.8 %) the AT tendon was in the slot in the navicular at the time of follow-up. It had become 'dis-inserted' from the groove in 48.2 % of cases. The AOFAS score (95.3 ± 4.7 vs 94.7 ± 5.3) and the talocalcaneal angle (5.39 ± 2.35 vs 5.76 ± 1.84) were not statistically significantly different whether the AT tendon was or was not sited in the navicular groove. All the patients were asymptomatic at follow-up.

***Conclusion*** The calcaneo-stop procedure is a simple, reliable, and minimally invasive procedure for the treatment of pediatric flexible flatfoot. It allows re-alignment of the talus and calcaneus, restoring a proper foot arch. Although the criteria of using the Young's suspension as an isolated procedure is very narrow, it can still be an effective procedure as shown in our algorithm. Ultrasound of the AT tendon demonstrated it's presence in the navicular groove at medium-long term (86.8 months ± 31.6, min follow up 8 months) in 57 % of cases. It was not possible to determine what was the minimum cut-off period for the AT tendon to stay in the navicular groove—ie 2 years.

***Significance*** A prospective serial ultrasound check might allow better characterisation of the role of the AT tenodesis is. The key to appropriate utilization of surgical procedures is a thorough understanding of the biomechanics of foot function, and a specific appreciation of the function of the tibialis anterior tendon.

## PP17

## Early diagnosis of cleidocranial dysplasia (CCD) with hip ultrasound

**Maurizio De Pellegrin**[1], Dario Fracassetti[1], Desiree Moharamzadeh[1]

[1]San Raffaele Hospital, Pediatric Orthopedic Unit, Milano, Italy

**Level-2**

**Hip/DDH/Legg-Calvé-Perthes/SCFE**

***Introduction*** Cleidocranial dysplasia (CCD) is a rare autosomal disorder primarily affecting bones formed by intramembranous ossification with a prevalence of less than 1:1.000.000. The aim of our study was to evaluate with US examination the hip morphology in its bony and cartilaginous components in CCD babies.

***Material and methods*** From 1985 to 2006, approximately 100,000 newborns' hips were ultrasonographically evaluated in our out-patient clinic dedicated to developmental dysplasia of the hip (DDH). Amongst these were three children (3 F) with CCD. Age range: 57–148 days. The dynamic US examination was performed with a linear 7.5 MHz probe according to Graf's method, in order to define the cartilaginous and bony coverage of the femoral head on a standard frontal plane scan. The total amount of femoral head coverage was determined using Morin's technique. The presence of the proximal femoral ossific nucleus was verified. Radiographs of the skull, spine and hands were taken along with conventional radiographs of the pelvis in order to further establish and confirm the diagnosis.

***Results*** All the hips showed a characteristic, incomplete shape of the acetabular roof. This appeared to be shortened due to the thickened cartilage component. The acetabular bony rim was rounded. It was impossible to identify the infero-medial margin of the ilium and, therefore, to measure the bony coverage of the femoral head with the alpha angle according to Graf, while the average value of the beta angle, an expression of the cartilaginous coverage, was 65°. The average coverage according to Morin was 58 % (average: 60 % in normal hips). The femoral ossific nucleus was present in one patient bilaterally, at an age of 3.3 months. In all cases X-ray evaluation of the pelvis confirmed the diagnosis of CCD. Radiographs of the skull, clavicles and spine confirmed the typical appearances of CCD.

***Conclusion*** The study highlights the presence of some ultrasonographic anomalies detectable in neonatal hips of babies with CCD.

***Significance*** Hip ultrasound allows the detection of typical altered bony and cartilaginous patterns in the acetabulum of CCD patients.

## PP18

## Radiological comparison of locking vs non-locking plates for temporary hemiepiphyseodesis: experimental study

**Dmytro Iershov**[1], Sergiy Khmyzov[1], Konstantin Romanenko[1], Viktor Rokutov[1]

[1]Sytenko Institute of Spine and Joint Pathology NAMS of Ukraine, Kharkiv, Ukraine

**Level-3**

**Basic science**

***Introduction*** Temporary hemiepiphysiodesis is a widely used technique to correct lower extremities angular deformity in childrens. Three types of plates are used for temporary blocking: 8-plate, hinge-plate and locking plate. They differ in biomechanical properties: non-locking plates allow angular deviation of the screw (while growing), locking plates provide angular stability of the screw. Due to their design the locking plates should provide faster deformity correction (blocking). There is a lack of comparative studies (except plate vs staple or among non-locking plates) devoted to the comparison of biomechanical properties and/or clinical results of different plate design application.

***Aim of the study*** To compare the rate of development of tibial varus deformity using locking vs non-locking plates by radiological analysis in rabbits.

***Material and methods*** We performed a prospective experimental study of twenty white rabbits (8 weeks old male, 2 kgs approx). Two groups (10 rabbits each) underwent a similar procedure: temporary medial proximal hemiepiphysiodesis of the right tibia with different plates for hemiepiphysiodesis (Group 1: non-locking plates, Group 2: locking plates). Animals were followed up weekly for next 8 weeks with AP view X-rays of the lower extremities. X-ray images were assessed according to Paley's protocol (MPTA, MAD determined and compared in both groups). Qualitative changes of the proximal tibial physis of the operated and non-operated side were compared. Statistical analysis was performed (t-test).

***Results*** We observed the progressive tibial varus deformity in both groups. Average MPTA on final X-rays: Group 1: 67.5 ± 3°; Group 2: 65.5 ± 3° ($p > 0.05$). The average rate of the deformity development: Group 1: 2.5°/week; Group 2: 2.8°/week ($p > 0.005$). No significant differences were observed between average MAD index in both groups. Similar qualitative radiographic changes were observed in the growth plate on the side of the block in both groups.
***Conclusion*** According to current study for temporary hemiepiphysiodesis, locking plates were not superior to the non-locking as far as they had not provided faster tibial varus deformity during follow up period, although we observed faster deformity development at the beginning of the study.
***Significance*** Using of the locking plate may be a suitable option for the angular lower limb deformity correction in children with limited growth potential (adolescent).

## PP19

## Locking vs non-locking plates for long bones length control (guided growth): experimental study

**Sergey Khmyzov**[1], Dmytro Iershov[2], Viktor Rokutov[3]

[1]Sytenko Institute of Spine and Joint Pathology, Kharkiv, Ukraine; [2]Sytenko Institute, Kharkiv, Ukraine; [3]Emergency Hospital, Dnepropetrovsk, Ukraine

**Level-3**

**Basic science**
***Introduction*** A temporary epiphysiodesis by bilateral physeal block with 8-plates is currently used to correct mild LLD in children. Recently several studies showed side effects of guided growth with 8-plates: low efficacy (Gaumétou E, 2015); secondary deformities (Lauge-Pedersen H, 2013). 8-plate design allows angular deviation of the screw (7°) which is the key issue in secondary deformity development. Hypothesis of the study—whether locking plates are superior to non-locking for temporary bilateral physeal blocking to control long bone length and prevention of secondary deformity development.
***Material and methods*** For experimental study we used 18, 8-week-old male white rabbits weighing approx. 2 kg. The animals underwent temporary bilateral physeal blocking of the right distal femur using non-locking (Group 1) and locking (Group 2) plates. Additional pins were inserted into both distal metaphyseal femur served as a landmark for the “Pin-Physis” distance measurement. The contralateral femur served as the control for each animal. Full length AP radiographs were performed on 3, 5 and 7 week. mLDFA (Paley’s protocol) and distance “Pin-Physis” in three points (on lateral, middle and medial aspects of the distal femoral physis) were measured. Qualitatively changes of the distal femoral physis were evaluated. Statistical analysis was performed (t-test).
***Results*** Progressive right femoral shortening was observed in both animal groups. In both groups there were no statistically significant changes of mLDFA on operated and control sides [at 7th week in Group 1: mLDFA (operated/control side) 97°/95°; in Group 2: 98°/96° ($p > 0.05$)]. Similar qualitative radiographic changes were observed in the distal femoral physis on the operated side in both groups. The “Pin-Physis” distances at 3, 5 and 7 weeks were progressively increased on the control side of both groups which indicates the normal distal femur longitudinal growth ($p < 0.05$). In Group 1 the “Pin-Physis” distances gradually changed from the 3rd up to the 7th week due to angular deviation of the screws in non-locking plates ($p < 0.05$). In Group 2 the “Pin-Physis” distances at 3, 5 and 7 weeks were constant on the operated side.
***Conclusion*** Bilateral physeal block using both locking and non-locking plates leads to progressive shortening of the bone. We did not observe secondary deformity of the distal femur in either group but, nevertheless, minor residual growth of the distal femoral physis blocked with non-locking plates was evident, especially in its midline point.
***Significance*** Using of locking plates provides more rigid blocking of physis and takes less time for LLD correction.

Open Access

This article is distributed under the terms of the Creative Commons Attribution 4.0 International License (http://creativecommons.org/licenses/by/4.0/), which permits unrestricted use, distribution, and reproduction in any medium, provided you give appropriate credit to the original author(s) and the source, provide a link to the Creative Commons license, and indicate if changes were made.

